# The Anchoring Effect in Study Time Allocation: Labor-in-Vain versus Labor-and-Gain

**DOI:** 10.3390/bs14070567

**Published:** 2024-07-04

**Authors:** Xiuya Li, Hui Xu, Yue Chu, Weihai Tang, Xiping Liu

**Affiliations:** Faculty of Psychology, Tianjin Normal University, Tianjin 300387, China; lixiuya1114@163.com (X.L.); xhpsych@126.com (H.X.); cypsy11@sina.com (Y.C.); twhpsy@126.com (W.T.)

**Keywords:** anchoring effect, self-paced study, metacognitive control, study time allocation, labor-and-gain effect

## Abstract

How to allocate study time is an important decision-making problem learners face. Research on this problem can help improve the learning performance of learners and provide guidance for teaching activities. This research aimed to explore the potential of anchors (prior information that may influence individual decision-making and judgment under uncertainty) as clues for study time allocation and examine the effectiveness of study time allocation under the influence of anchors. Sixty-two Chinese university students (*M*_age_ = 21.21, *SD* = 1.74; 44 females) studied 20 word pairs under self-paced learning instructions. These instructions either set a high anchor (i.e., the typical participant spent 15 s learning each pair) or a low anchor (i.e., the typical participant spent 5 s learning each pair) for study time. After a brief distraction phase, participants took a cued recall test. The results showed that the higher the anchor value, the longer the corresponding study time, and the longer the study time, the better the memory performance. These results reveal that there is both an anchoring effect and a labor-and-gain effect in self-paced study time allocation. This study extends the range of observable anchoring effects and provides important information on allocating study time effectively.

## 1. Introduction

### 1.1. Study Time Allocation

Study time allocation refers to the process through which learners allocate their time and mental resources to different study items or tasks to achieve optimal learning, reflecting individual management and control of mental resources [1,2]. Study time allocation is an important indicator of metacognitive control [3], including item selection and self-paced study time [4,5,6,7].

The typical study time allocation paradigm usually includes a self-paced study phase and a recall test phase. During the self-paced study phase, participants are exposed to study items such as words, word pairs, or translations, and they paced their study time for each item. In this process, the time participants allocate to each item during the self-paced study phase represents their study time allocation. Following the study phase, a recall test is conducted to evaluate the individual’s memory performance on the studied items [5,8].

Study time allocation is effective when it improves late retention for items that receive more study time [5]. If items studied for longer durations demonstrate better memory performance, we define it as the labor-and-gain effect; conversely, if items studied for longer durations do not exhibit better memory performance, we define it as the labor-in-vain effect. A finding reported by Nelson and Leonesio [9] suggested that the accuracy-emphasis group spent more time studying than the speed-emphasis group, yet there was no retention difference between the two groups. This implies that allocating more study time does not necessarily lead to better memory performance. Peng and Tullis [10] employed the honor/dishonor paradigm [11,12] to investigate the impact of divided attention on restudy selection. Under both the divided and full attention conditions, participants were instructed to select half of the items to restudy. Subsequently, researchers provided them with additional study time for restudy. Each participant restudied half of the items they had selected (honoring their selection) and half of the items they had not selected (dishonoring their selection). Recall performance for honored and dishonored choices was then compared across different attention conditions. The findings revealed that under divided attention, there was no difference in recall performance between honored and dishonored restudy choices. However, under full attention, honoring participants’ restudy choices yielded greater cued recall than dishonoring their restudy choices. This indicates that divided attention impairs the effectiveness of restudy selection.

How do learners allocate their study time? Since the 1960s, researchers have investigated this question for nearly six decades, successively proposing the discrepancy reduction model [13], the hierarchical model of self-regulation study [7], and the region of proximal learning model [14] to elucidate the underlying mechanisms of study time allocation. Although these three models have notable differences, they share a common core point: item difficulty drives study time allocation. As research has progressed, scholars have gradually recognized that item difficulty may not be the sole factor guiding study time allocation [15], and other factors such as reward structure [16], habitual responses [4,17,18], and individual differences [19,20,21] also exert influence. Based on this, Ariel et al. [16] proposed the agenda-based regulation (ABR) model, which challenges the traditional theoretical framework of difficulty-based study time allocation, emphasizing the core role of agendas in study regulation and control. According to this model, self-paced learning is goal-oriented. To maximize goal achievement, learners comprehensively analyze various possible subjective and objective factors to construct an agenda that guides the allocation of study time and execute this agenda during the study phase [17,22].

In addition, the ABR model also acknowledges some irrational factors, such as habitual responses, which can capture the allocation of learning time and override the influence of agenda use. For example, Ariel et al. [23] examined the impact of habitual responses on item selection. In their study, native Arabic speakers and native English readers were required to learn word pairs from each difficulty level (easy, moderate, and difficult) within 15 s. The cue words of these pairs are simultaneously presented from left to right on the computer screen and the order of difficulty was counterbalanced across trials. The difficulty label of each item was presented above each cue. While the optimal strategy would be to prioritize learning the easiest item on each trial, most participants failed to construct and execute this agenda. Instead, regardless of the difficulty, native Arabic speakers prioritized learning items at the right position of each array, and native English readers prioritized learning items at the left position of each array. This study illustrates that habitual responses can undermine learners’ agenda use and bias study selection.

Therefore, it is essential to examine whether study time allocation is rational and whether it is influenced by extraneous factors beyond the agenda. This exploration not only expands the ABR model but also provides valuable information for learners to efficiently utilize study time, optimize study time allocation strategies, and maximize the achievement of learning goals.

### 1.2. Anchoring Effect in Metamemory

The anchoring effect refers to a bias phenomenon in which the results of individual decision-making and judgment are biased towards previously presented information under uncertainty [24]. For example, in a study conducted by Tversky and Kahneman [24], participants were asked to judge whether the proportion of African countries in the United Nations is higher or lower than an arbitrary number generated by a wheel of fortune (high anchor: 65%; low anchor: 10%). Subsequently, each participant made an absolute estimate. The results demonstrated that participants in the high-anchor condition made higher estimations (45%) than those in the low-anchor condition (15%). Anchoring effects provide important empirical evidence for limited rationality models of decision-making [25].

The selective-accessibility theory has been proposed to explain anchoring effects. According to the selective-accessibility theory, contacting an anchor prompts individuals to construct a mental model in which the target value is equal to the anchor value to test whether the target value is consistent with the anchor value. This semantically activates target features that are consistent with the anchor value, leading to the accessibility of this information [26,27,28].

The anchoring effect is a common phenomenon in human decision-making and judgment [25]. Many empirical studies have demonstrated its existence in very disparate fields, such as judicial decisions [29,30], valuation judgments [31], and even cognitive tasks [32,33]. However, a few studies have explored whether the anchoring effects exist in the metacognitive domain.

England and Serra [34] demonstrated that informative anchors, closely related to judgment situations, can bias metacognitive monitoring. In their study, participants in the high-anchor condition were informed that the subsequent memory task was pretty easy, and most learners recalled approximately 90%, while those in the low-anchor condition were informed that the subsequent memory task was pretty difficult, and most learners recalled approximately 10%. After exposure to anchoring information, participants were asked to make judgments of learning (JOLs) for each item to predict the likelihood that each item would be recalled in the subsequent test. It was observed that JOLs in the high-anchor condition were significantly higher than those in the low-anchor condition, even though participants learned the same words under both high- and low-anchor conditions.

Yang et al. [35] extended the findings regarding informative anchors to uninformative anchors, confirming that uninformative anchors, which are randomly selected and should be ignored by a rational judge, can also bias JOLs. Later, Ikeda [36] further demonstrated that the influence of uninformative anchors on JOLs is robust and cannot be eliminated by prior learning experience.

Overall, the existence of anchoring effects in metamemory monitoring has been demonstrated by many empirical studies. However, do anchors influence metamemory control? This issue was first explored by Yang et al. [35]. In their experiment, participants were presented with word pairs and given 5 s to study each pair. Subsequently, they were asked to judge whether the likelihood of recalling each word pair in the memory test was higher or lower than an anchor value ranging from 10% to 90%. Finally, participants made JOLs and were asked whether they wanted to restudy this pair after the initial study phase. The results revealed that although there was no significant difference in recall performance, the proportion of pairs selected by participants for restudy decreased as the anchor value increased. This suggests that anchoring effects may also exist in study time allocation.

### 1.3. Limitations of Previous Research

Although Yang et al. [35] confirmed that anchors can potentially influence restudy selection, further work is necessary to replicate this finding. Furthermore, their study has some limitations. First, participants were required to make a JOL after encountering an anchor and before selecting a restudy item. According to the monitoring-affects-control model, metacognitive control is closely related to metacognitive monitoring. Learners typically regulate and control their learning behaviors based on metacognitive monitoring [9,37]. Specifically, a high JOL rating may signal to the learner that restudy is unnecessary, while a lower JOL rating may prompt the learner to restudy. It is therefore necessary to consider whether learners’ study time allocation would still be influenced by anchors in the absence of an explicit monitoring requirement. This question deserves serious consideration because restudy selection after making a JOL may be directly affected by the JOL itself, rather than the anchors. More importantly, in authentic learning settings, learners do not consciously monitor their learning activities. Therefore, exploring whether anchors influence individuals’ study time allocation directly becomes an important issue for further study.

Second, study time allocation generally includes item selection and self-paced study time [4]. While Yang et al. [35] focused on investigating the influence of anchors on the restudy selection, they did not explore the effect of anchors on self-paced study time. Ikeda et al. [21] found that learners’ beliefs about intelligence affect the restudy selection but not study time allocation. This suggests that item selection and study time allocation may be driven by different cognitive processes. Therefore, examining whether anchors affect self-paced learning time is the second issue addressed in this study.

Finally, study time allocation may be effective (labor-and-gain effect) or ineffective (labor-in-vain effect). However, in the study by Yang et al. [35], participants studied each word pair at a fixed presentation rate throughout the study phase. In addition, although participants had previously selected restudy items, they were not allowed to restudy them before the final recall test. Accordingly, it is unclear whether the differences in the study time allocation induced by anchors could subsequently impact participants’ memory performance. Therefore, exploring whether the study time allocation influenced by anchors is effective becomes the third issue that requires further research.

### 1.4. The Current Study

Exploring the impact of anchoring information on study time allocation has practical and theoretical significance in current metacognitive research. In daily learning and life, learners can be exposed to a variety of information from the external environment, such as advice from teachers, the performance of peers, or even incidentally encountered cues, all of which may serve as anchors. Therefore, gaining a better understanding of how anchoring information affects metacognitive monitoring may provide valuable insights into metacognitive control in coping with such situations. In addition, as mentioned earlier, the ABR model suggests that some irrational factors may exert a great influence on study time allocation, overriding the use of agendas. However, previous research has primarily focused on investigating the role of habitual responses. Therefore, the current study can expand the ABR model by confirming the anchoring effect on study time allocation.

In the current study, we investigated whether participants demonstrated anchoring effects in their study time for a list of word pairs to be remembered. Specifically, we presented participants with anchoring information before learning. The impact of anchors on study time allocation was examined by comparing participants’ study time between high-anchor and low-anchor conditions. According to selective-accessibility theory, we expected study time allocation to demonstrate anchoring effects. Specifically, we expected participants in the high-anchor condition to spend more time studying than those in the low-anchor condition.

In addition, we also focused on the effectiveness of self-paced study time. If the labor-and-gain effect exists, the memory performance of items with a long study time could be better than those with a short study time. If the labor-in-vain effect exists, there should be no significant difference in memory performance between the items with a long study time and those with a short study time.

## 2. Materials and Methods

### 2.1. Design

The experiment adopted a between-subjects design in which the independent variable was the anchor condition (high-anchor vs. low-anchor), and the dependent variables were study time and final recall.

### 2.2. Participants

Sixty-two college students were recruited to take part in this study for RMB 5, aged between 18 and 25 years old (M_age_ = 21.21, SD = 1.74; 44 females). All participants were native Chinese speakers. An ABBA design was employed to assign participants to different anchor conditions. Among them, 31 participants (M_age_ = 21.16, SD = 1.68; 22 females) were assigned to the high-anchor condition, while the remaining 31 participants (M_age_ = 21.26, SD = 1.83; 22 females) were assigned to the low-anchor condition. All participants provided informed consent before the experiment. To estimate the statistical power of this study, we conducted a post-hoc sensitivity analysis using G*Power 3.1. The sensitivity analysis indicated that with our sample size, we had sufficient power (0.90) to detect an effect size of d = 0.72 with an alpha level of 0.05. This provides confidence that our study design is adequately powered to detect meaningful effects.

### 2.3. Materials

The study list consisted of 24 unrelated Chinese noun word pairs, of which 4 were used for practice and the others were used for the formal experiment. In the pilot experiment, 22 college students (M_age_ = 25.68, SD = 2.77) were recruited to assess the difficulty and familiarity of each word on a 5-point scale. The results showed that the difficulty of these words ranged from 4.27 to 4.95 (M = 4.64, SD = 0.15), and the familiarity ranged from 4.36 to 4.91 (M = 4.75, SD = 0.13). Then the students rated these word pairs in terms of their relatedness on a 5-point scale, which showed that the relatedness of these pairs ranged from 1.23 to 1.77 (M = 1.44, SD = 0.15).

### 2.4. Procedure

**Figure 1:**
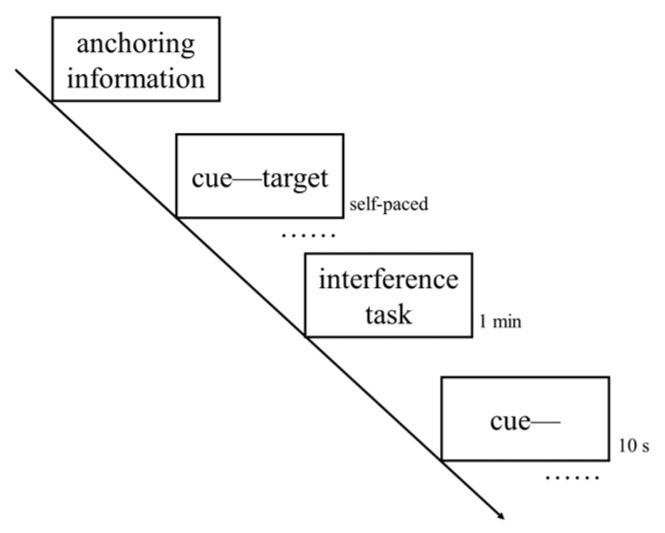
The procedure of the experiment.

To help them become familiar with the experimental procedure, participants completed a short practice. The whole experiment mainly included four phases, as shown in Figure 1 and outlined below.

Anchors exposure phase. Referring to England and Serra [34] and Yang et al. [35], the anchor conditions were manipulated by using instructions. In the high-anchor condition, participants were informed that “According to our previous studies, the time for college students to study each word pair was an average of 15 s in the following memory task”. In the low-anchor condition, participants were informed that “According to our previous studies, the time for college students to study each word pair was an average of 5 s in the following memory task”. The anchor values setting was based on Jiang et al. [38], in which college students spent 8.14~13.05 s studying each Chinese word pair.

Study phase. Participants studied a total of 20 word pairs for a later memory test and were informed that their goal was to remember as many pairs as possible. At the beginning of each trial, a fixation point appeared in the center of the computer screen for 1 s, followed by a word pair presented in black 48-point Microsoft YaHei font. Participants studied each pair, which was randomly presented one at a time in a self-paced manner, and were required to press the space bar to start the next trial when they had completed studying. During this process, the computer recorded the study time of the participants for each pair.

Distractor phase. Participants were asked to complete a 1-min mathematical operation task.

Test phase. All 20 cue words (the first word of each pair) were randomly presented, one by one. Participants were instructed to recall each corresponding target word (the second word of each pair) and report it within 10 s.

Finally, to assess participants’ perceptions of high- and low-anchor values, participants were asked to answer the following two questions: (1) Do you think the statement that “According to our previous studies, the time for college students to study each word pair was an average of 5/15 s in the following memory task” was reliable? (2) Do you think the statement “According to our previous studies, the time for college students to study each word pair was an average of 5/15 s in the following memory task” affected your study time? These two questions were rated on a 5-point scale.

All experimental procedures were approved by the institutional ethics committee and conducted in accordance with the Declaration of Helsinki.

## 3. Results

### 3.1. Participants’ Perceptions of High- and Low-Anchor

An independent-sample *t*-test was conducted on the question scores of the participants in the different anchor conditions. The results showed that the participants in the high-anchor condition (M = 5.19, SD = 2.21) and those in the low-anchor condition (M = 5.55, SD = 2.58) perceived the anchoring information as equally reliable, *t*(60) = −0.58, *p* = 0.563, Cohen’s d = −0.15. In addition, participants in both the high-anchor condition (*M* = 5.42, SD = 2.71) and the low-anchor condition (M = 4.97, SD = 2.46) considered that the anchoring information had a moderate effect on their study time, *t*(60) = −0.69, *p* = 0.494, Cohen’s d = 0.18. This analysis suggests that participants’ perceptions of high- and low-anchor values were comparable.

### 3.2. Effect of Anchors on Study Time

**Figure 2:**
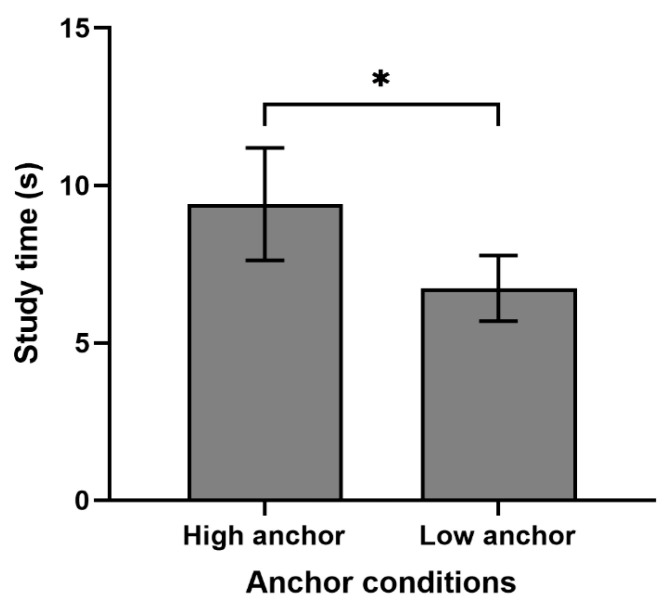
Study time for the high- and low-anchor conditions (error bars represent 95% confidence intervals). Note. * *p* < 0.05.

An independent-sample *t*-test was conducted with the anchor condition as a between-subjects variable and study time as a dependent variable to examine the effect of anchors on the study time allocation. The results showed that the study time in the high-anchor condition (M = 9.40, SD = 4.87) was significantly longer than that in the low-anchor condition (M = 6.74, SD = 2.85), *t*(60) = 2.63, *p* = 0.011, Cohen’s d = 0.67 (see Figure 2). This suggests that participants allocated more time to study in the high-anchor condition.

### 3.3. Effect of Anchors on Cued-Recall Performance

**Figure 3:**
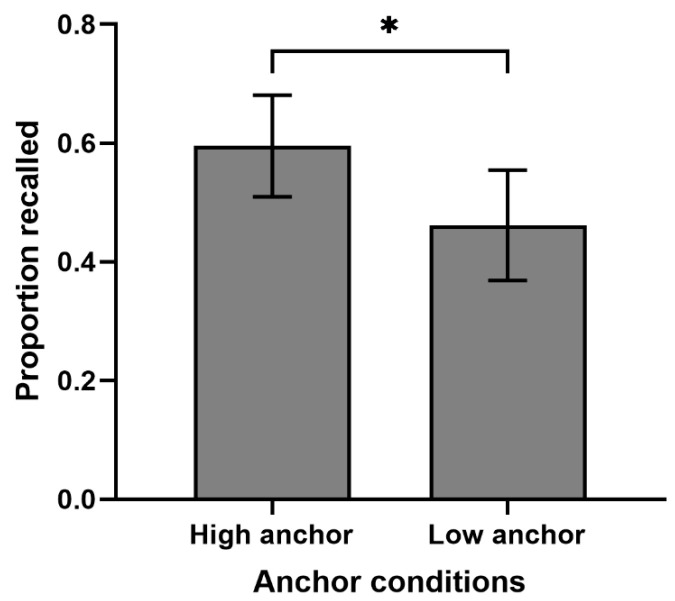
Cued-recall performance for high- and low-anchor conditions (error bars represent 95% confidence intervals). Note. * *p* < 0.054.

An independent-sample *t*-test was used to compare the recall proportion of word pairs in high- and low-anchor conditions. It was found that the recall proportion in the high-anchor condition (M = 0.60, SD = 0.23) was significantly higher than that in the low-anchor condition (M = 0.46, SD = 0.25), *t*(60) = 2.17, *p* = 0.034, Cohen’s d = 0.55 (see Figure 3). This indicates that the longer the study time, the higher the cued-recall performance.

## 4. Discussion

Using the standard study time allocation paradigm, we examined the differences in learners’ study time and memory performance by manipulating the anchor conditions. The results showed that higher anchor values led to longer study time, resulting in improved memory performance. This demonstrates both the anchoring effect and the labor-and-gain effect in self-paced study time allocation.

Yang et al. [35] found that the number of restudy items selected by participants in the high-anchor condition was significantly higher than that in the low-anchor condition, suggesting that learners in the high-anchor condition tended to allocate more time for additional learning. This is consistent with the results of the present research. However, Yang and colleagues did not clarify whether study time allocation is influenced by anchors or constrained by JOLs. In this study, the effects of JOLs and anchors were separated, illustrating that even without making JOLs, anchors still affect study time allocation. It is important to note that although participants were not asked to make conscious monitoring judgments in this study, there may have been unconscious monitoring during the learning process. Anchors were likely to affect their unconscious monitoring of the learning materials by changing their beliefs, which further affected study time allocation.

Past theories on self-paced study have emphasized the influence of item difficulty on study time allocation, suggesting that the differences in difficulty lead to either less or more study time, as confirmed by numerous studies [14,15,39]. However, it is difficult to interpret the results of this study within the framework of these theories. This is because it was observed in this study that participants in the high-anchor condition allocated more study time than those in the low-anchor condition, even though both groups learned the same words. It should be noted that our main conclusion does not deny the role of the above three theoretical models in explaining self-paced study time allocation but rather indicates that item difficulty is not the only factor influencing study time allocation. Other factors, such as anchors, may also influence study time allocation. Therefore, the current study provides empirical support for the ABR model.

This phenomenon can be explained by the combination of the ABR model and selective-accessibility theory. In the present study, the learner’s goal was to memorize as many pairs as possible. To achieve this goal, learners may analyze various factors such as item difficulty and task constraints to construct an agenda that guides study time allocation. In this process, the anchors encountered before the study can act as cues that learners consider when developing their agenda. According to the selective-accessibility theory, these anchors induce learners to construct a mental model to test the consistency between the target value and the anchor value, thereby activating information consistent with the anchor value to be accessible. In other words, anchors prompt learners to consider subjective and objective factors that might confirm that they need 5 s (low-anchor)/15 s (high-anchor) to remember each word pair.

In addition, this study also found that differences in the study time allocation between the high- and low-anchor conditions further resulted in differences in recall performance. Specifically, learners in the high-anchor condition could recall more pairs compared to those in the low-anchor condition. This indicates that participants benefit from additional learning, leading to the labor-and-gain effect. Note, however, that not all studies have found the labor-and-gain effect. Nelson and Leonesio [9] proposed that there are two possible boundary conditions for the labor-in-vain effect, one of which is the learning activities carried out by individuals. According to this view, ineffective learning activities potentially lead to the labor-in-vain effect. Conversely, the labor-and-gain effect may occur after effective learning activities. Therefore, it can be inferred that learners in the high-anchor condition engage in relatively effective learning activities over a longer study time.

The findings have implications for education and teaching practice. Information about past peer performance (i.e., anchor) can serve as cues for study time allocation. This implies that learners are susceptible to the external environment when allocating study time, so educators should be cautious when providing students with information such as the performance of other learners and standards. Furthermore, anchor-based study time allocation may lead to the emergence of the labor-and-gain effect. Therefore, before students engage in self-paced learning, educators can appropriately provide some high-anchor information to motivate students to invest more time in relatively effective learning.

Although this study may deepen our understanding of human learning activities and provide effective guidance on how to improve self-paced study, there were also some limitations. First, anchors can be divided into self-generated anchors and externally provided anchors according to the source [40,41,42]. Self-generated anchors are based on one’s own knowledge and experience, such as the learner’s beliefs. Externally provided anchors are provided by the external environment or others, such as the peer performance information used in this study. How these two types of anchors interact and collectively influence study time allocation could be explored in the future. Second, the age range of the participants in this study focused on 18–25 years old. Considering that the ability of self-paced study time allocation tends to improve with age [43,44,45], future developmental studies must be conducted to further explore whether age affects the anchoring effect in study time allocation. Finally, future research should also examine possible factors that may enhance or attenuate the anchoring effect in study time allocation. For example, with the accumulation of learning experience, do anchors continue to influence study time allocation? As cognitive load increases, does study time allocation become more dependent on anchors? Are field-dependent learners more susceptible to anchors compared to field-independent learners?

## 5. Conclusions

In summary, study time allocation is indeed sensitive to the anchors encountered before learning, which complements and expands the ABR model. Furthermore, the differences in anchor-based study time allocation further led to changes in recall performance. Thus, there is both an anchoring effect and a labor-and-gain effect in self-paced study time allocation.

## Data Availability

The dataset is available upon request from the authors. All data included in the current study can be obtained from the corresponding author upon reasonable request.
